# Psychosocial interventions for rehabilitation and reintegration into daily life of pediatric cancer survivors and their families: A systematic review

**DOI:** 10.1371/journal.pone.0196151

**Published:** 2018-04-19

**Authors:** Mona Leandra Peikert, Laura Inhestern, Corinna Bergelt

**Affiliations:** Department of Medical Psychology, University Medical Center Hamburg-Eppendorf, Hamburg, Germany; York University, UNITED KINGDOM

## Abstract

**Background:**

The survival rate of childhood cancer patients increased over the past decades. However, even after successful treatment the transition back to normalcy is often a major challenge for the whole family. Therefore, this study aims to provide an overview of psychosocial interventions for childhood cancer survivors and their families in the first years after the end of cancer treatment.

**Methods:**

We conducted a systematic review following the PRISMA Checklist (Preferred Reporting Items for Systematic Reviews and Meta-Analyses; PROSPERO registration number: CRD42017059782). In November 2016 and September 2017, we searched the databases CINAHL, MEDLINE, PSYNDEX, and Web of Science. We included studies investigating psychosocial interventions for childhood cancer survivors diagnosed under the age of 21, their family members or the family as a whole. Further, we summarized the study characteristics and conducted a narrative synthesis of the results. Finally, we assessed the study quality with the Effective Public Health Practice Project Quality Assessment Tool.

**Results:**

We identified a total of 8215 records based on our database searches and 17 additional records through hand searches. We included 33 articles in the qualitative synthesis. Most of the studies described interventions for the cancer survivor (n = 15). Nine studies investigated interventions for the whole family, and two studies interventions for siblings. The interventions mainly take place in an outpatient group setting (n = 15). Overall, most of the studies reported a significant psychosocial benefit of the interventions. However, the quality of the included studies was limited.

**Conclusion:**

In summary, we identified a broad range of different interventions and thus could give a comprehensive overview of existing interventions for childhood cancer survivors and their families. However, there is a necessity for high quality studies. The results may help to optimize health care services that support families with the re-entry into daily life.

## Introduction

A childhood cancer diagnosis turns the lives of patients and family members upside-down. The incidence rates range from approximately 120 per million to more than 170 per million in developed countries [1]. Diagnostic and treatment methods improved a lot in the past decades. The five-year survival rate for childhood cancer in developed countries increased from 65% in the 1980s to approximately 80% nowadays [1], which leads to a growing population of childhood cancer survivors.

However, even despite a complete remission of childhood cancer, the transition back to normalcy is often long and difficult [2]. After the completion of treatment, childhood cancer patients report significantly lower physical well-being than their healthy peers [3]. The psychological well-being of cancer patients seems to increase after diagnosis [4–6]. Nevertheless, pediatric cancer survivors are at risk for developing stress-related mental disorders and are highly distressed [7–9]. Even more than seven years after diagnosis, a relevant proportion of adult survivors of childhood cancer report clinically relevant psychological distress symptoms whereof some fulfil diagnostic criteria of a post-traumatic stress disorder (PTSD) [10].

Notably, also the patient’s family as a system and the family members individually can suffer from negative psychosocial consequences of childhood cancer [11, 12]. After the first shock caused by the cancer diagnosis, parents have to adapt to the new situation, their new roles and responsibilities [13]. Especially an active treatment status is associated with low parental quality of life and high psychological distress [6, 11, 14]. Still, post-traumatic stress symptoms or PTSD can also occur in parents after successful treatment of childhood cancer [15] and siblings of childhood cancer patients are also affected [16]. Siblings have to fulfil new roles and responsibilities within their families [17]. Further, siblings often get less attention by their parents than patients and in some families the extended family cares for the siblings [12]. Furthermore, they are emotionally burdened, report a poor quality of life and school difficulties can occur as a consequence of the cancer disease of their sibling [17]. Some siblings also report cancer-related traumatic stress reactions or fulfill the criteria for PTSD [18, 19]. During the first two years after diagnosis, the burden of siblings seems to be highest [17]. In summary, childhood cancer seems to be a family challenge that goes far beyond cancer treatment.

In addition to medical follow-up, evidence-based interventions are required to alleviate negative effects in childhood cancer patients, their parents and siblings [20, 21]. In the first years after the long and intensive cancer treatment, the physical and mental rehabilitation and reintegration into daily life of survivors and their family members (e.g. school life, working life, social life) are particularly important. Various interventions for childhood cancer survivors and their family members were evaluated and summarized in prior reviews. Some of these reviews included interventions on psychosocial and neurocognitive outcomes as well as health promotion solely for childhood cancer survivors [22–25] and a further review refers to psychosocial interventions solely for siblings [26]. Other reviews included studies on interventions for families of patients and survivors [27–33]. However, previous reviews often included a wide range of studies with regard to the aim of the intervention (psychosocial to neurocognitive interventions, e.g. [22]), the included diseases (not only cancer, e.g. [28]), the time since diagnosis (newly diagnosed patients to survivors, e.g.[31, 33]) or the age of the childhood cancer survivors (children and adults up to 56 years, e.g. [22]).

The burden and needs of affected families change over time [12] and depending on their current age, patients face different challenges and may have specific developmental needs (e.g. keeping up in school, development of autonomy). In this systematic review, we investigate interventions for childhood cancer survivors and their family members with a narrower focus in terms of type of intervention, diagnoses, time since cancer treatment, and age of the survivor. The review aims to provide an overview of psychosocial interventions for childhood cancer survivors diagnosed before the age of 21 and their family members in the first years after the end of acute cancer treatment.

## Methods

We developed our research questions in concordance with the PICO criteria [34]. Thus, we specified the participants (P), interventions (I), and outcomes (O) in our research questions. We included all studies reporting interventions independent of the presence or absence of a comparison group. Therefore, we did not specified the comparison (C) in our research questions. This systematic review addresses the following two research questions:

1. Which psychosocial interventions for rehabilitation and reintegration into daily life of pediatric cancer patients and their families after the end of acute cancer treatment were evaluated and published?
2. What are the effects of these interventions on psychosocial outcomes in the family members?

The reporting of this systematic review follows the PRISMA Checklist (Preferred Reporting Items for Systematic Reviews and Meta-Analyses) to conduce to transparency and completeness (S1 Table) [35, 36]. We published a review protocol in the International prospective register of systematic reviews (PROSPERO; www.crd.york.ac.uk/PROSPERO/; PROSPERO registration number: CRD42017059782; S1 File).

### Eligibility criteria

The eligibility criteria address three aspects: Study characteristics, participants and intervention. The inclusion criteria for study characteristics were: (1) Language English or German, (2) full text accessible, (3) no conference proceedings, (4) article published in a peer-reviewed journal, (5) primary research (no study protocols or intervention descriptions) and (6) not only qualitative research. The participants in the studies had to meet the following inclusion criteria: (1) Cancer patients and/or their family members, (2) the patient was diagnosed with cancer before the age of 21 and (3) no primary focus on palliative cancer patients. Finally, the inclusion criteria for the evaluated interventions were: (1) Psychosocial intervention (not primary pharmacological, neurocognitive or educational interventions), (2) cancer specific intervention, (3) child-, family- or parent-focused intervention and (4) intervention directed primarily at patients or family members after the end of cancer treatment to five years later (no primary focus on long-term survivors or patients receiving acute cancer treatment).

### Search

We searched the databases CINAHL, MEDLINE (via OVID), PSYNDEX (via OVID) and Web of Science in November 2016 and updated our search in September 2017. There were no restrictions regarding the publication period. The search strategy included the following terms (example OVID): (intervention* OR counseling OR support* OR therap* OR program* OR measure*) AND (psych* OR social) AND (p?ediatric OR child* OR adolescen*) AND (cancer OR oncolog* OR neoplasm OR malignan* OR tumo?r* OR leukemia OR lymphoma) AND (aftercare OR aftertreatment OR posttreatment OR off treatment OR rehabilitation OR reintegration OR surviv*) AND (child* OR patient* OR survivor* OR mother* OR father* OR parent* OR famil* OR sibling*). Additionally, hand searches of reference lists of relevant papers as well as included studies were conducted and reference lists of previous relevant reviews were checked [20–33, 37–42].

### Study selection

As a first step, duplicates were removed and the first author (MLP) screened the titles. Second, the first author screened the abstracts of the eligible studies. Third, the first author and an independent member of the research team (LI) screened the full texts. Disagreements between the raters were solved by discussion.

### Data collection and quality assessment

After the full text screening, two independent members of the research team (MLP and LI) extracted data from the included studies using a data extraction form. The data extraction form included information on study characteristics (authors, year, title, language, country), methods (study design, sample size, patient’s age at diagnosis, patient’s age at intervention, time since diagnosis, time since end of cancer treatment) and investigated interventions (name of intervention, target group, setting, psychosocial outcomes, effects of the intervention).

Additionally, both members of the research team assessed the quality of the included studies independently with the Effective Public Health Practice Project Quality Assessment Tool (EPHPP Quality Assessment Tool) [43, 44]. The EPHPP Quality Assessment Tool assesses six components: Selection bias, design, confounders, blinding, data collection methods, and withdrawals and drop-outs. Ratings (strong, moderate, weak) are applied on all six components. Additionally, studies receive a final grade. Studies with no weak ratings and at least four strong ratings on the six components receive the final grade strong [43]. Studies with two or more weak ratings receive the final grade weak. All other studies are considered moderate. Disagreements between raters were solved by discussion.

### Data synthesis

As anticipated, a quantitative data synthesis was not feasible due to the clinical, methodological and statistical heterogeneity of the studies. Therefore, we conducted a narrative synthesis as predefined in our study protocol [45]. We referred to the recommendations of the Cochrane Consumers and Communication Review Group on data synthesis and analysis [46].

## Results

### Study selection

**Fig 1:**
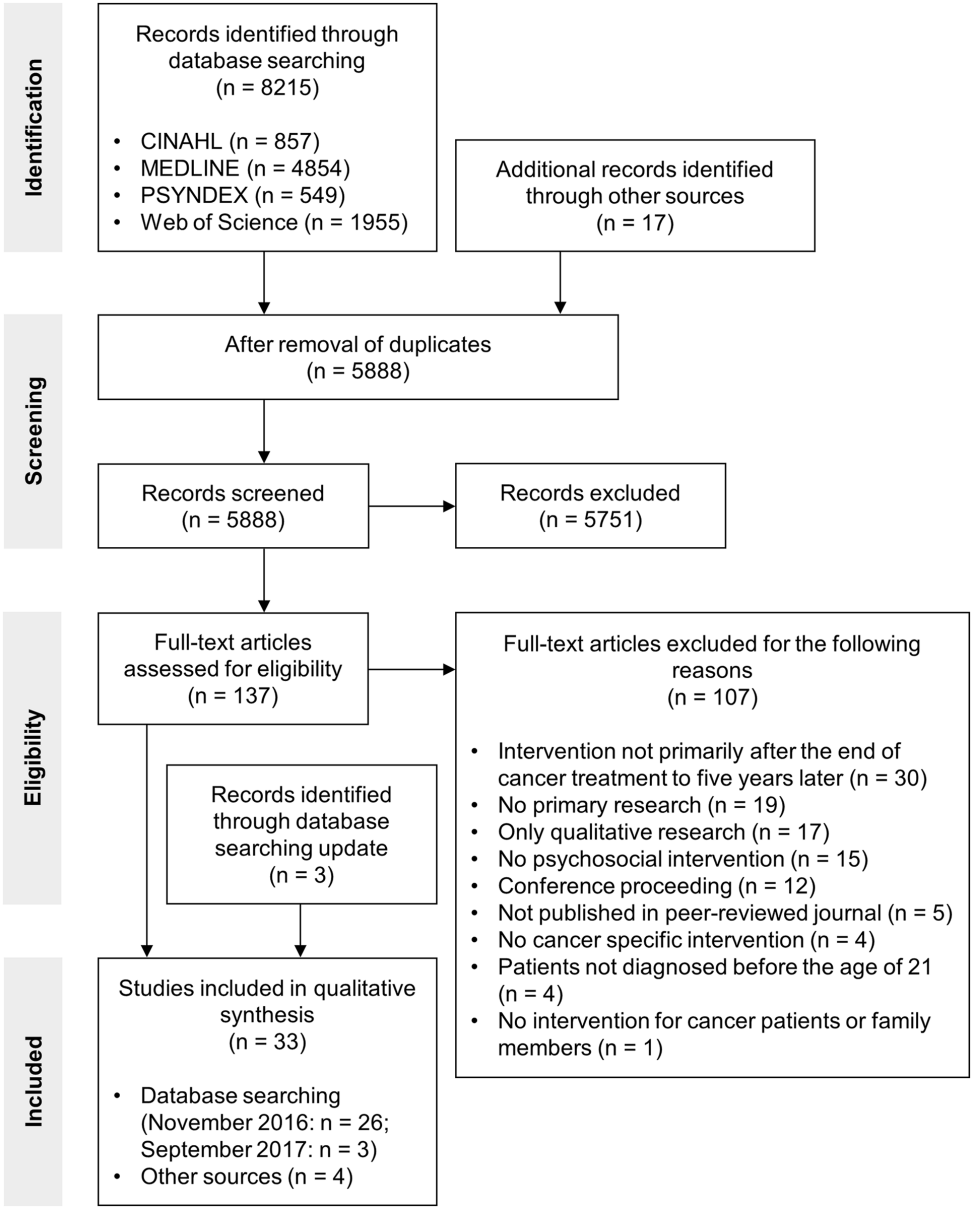
PRISMA flow diagram of the systematic literature search.

We identified a total of 8215 potentially relevant records through our database searches in November 2016. An additional 17 records were identified through hand searches (e.g. reference lists of relevant papers and previous reviews). After the removal of duplicates, 5888 titles and in a second step, 251 abstracts were screened. After this screening process, we excluded 5751 records. The remaining 137 full-texts were assessed for eligibility. First, 30 articles were included in the qualitative synthesis. The main reasons for the exclusion of 107 full-text articles were: Intervention not primarily conducted after the end of cancer treatment to five years later (n = 30), no primary research (n = 19), only qualitative research (n = 17), no psychosocial intervention (n = 15), and conference proceeding (n = 12). Three studies were identified through our search update in September 2017 and therefore we included a total of 33 studies in the qualitative synthesis. The study selection process is illustrated in the PRISMA flow diagram in Fig 1.

### Study characteristics and synthesis of results

In the 33 included papers, 24 interventions were evaluated. Four interventions were each investigated in two or more papers (Group Social Skills Intervention Program [47–50], Surviving Cancer Competently Intervention Program [51, 52], Quality of Life in Motion [53, 54], Family oriented rehabilitation [55–59]). However, the samples or the focuses of the articles differ and some interventions have been revised over time. Therefore, we decided to include all papers independently in the descriptive analyses of the study characteristics and results. Table 1 provides an overview of the relevant study information.

**Table 1:** Overview of the included studies (N = 33).

Name of the intervention	1. Study; 2. Country	1. Study design (groups; measurement time points); 2. Sample size	1. Age at diagnosis; 2. Time since diagnosis; 3. Time since end of treatment	Target group	Setting	Psychosocial effects of the intervention
Boys’ Group and Parents’ Group	1. Die-Trill et al., 1996 [60]; 2. USA	1. Uncontrolled cross-sectional intervention study (boys and parents; after final group session); 2. n = 8 boys, n = 9 parents	1. N/A; 2. M = 4 years (SD = 3.5); 3. N/A	Boys between 10 and 14 years of age, treated for a brain tumor; Their parents	Boy’s group: 8 consecutive 1-hour sessions per week, extended to 16 sessions on request; Parents’ group; Feedback sessions after 8th session and final sessions	Only assessment of satisfaction with the group interventions; No psychosocial outcome parameters assessed
Camp Little Red Door	1. Dawson et al., 2012 [61]; 2. USA	1. Uncontrolled intervention study (intervention group; pre, post, 3-month follow-up); 2. n = 29 children	1. N/A; 2. N/A; 3. N/A	8 to 18 year-olds, cancer diagnosis, post traditional medical treatment; Their siblings	Three-day camp for the oldest camper group precedes week-long camp for all campers	Increased independence, social skills and self-esteem from pre to post, only significant for self-esteem
Camp Okizu	1. Wu et al., 2011 [62]; 2. USA	1. Uncontrolled cross-sectional intervention study (participating families; post camp); 2. n = 89 families (n = 78 mothers, n = 9 fathers, n = 56 children with cancer, n = 73 siblings, 8 of whom were bereaved)	1. M = 6.9 years (SD = 4.2); 2. N/A; 3. 89.3% off treatment, 10.7% on treatment	Children and adolescents with cancer, siblings of children with cancer, and their parents	7 1-week residential camp sessions during the summer, 3 sessions for children with cancer, and 4 sessions for siblings	Only assessment of satisfaction with the camp; No psychosocial outcome parameters assessed
Cognitive and Behavioural Therapy (CBT) Intervention	1. Poggi et al., 2009 [63]; 2. Italy	1. Non-randomized controlled intervention study (intervention group, control group; pre, post); 2. n = 40 patients (n = 17 intervention group, n = 23 control group)	1. M = 5.53 years (SD = 3.78); 2. N/A; 3. N/A	Children and adolescents surviving brain cancer, 4 to 18 years old	Treatment based on cognitive and behavioral therapy, 4 to 8 months with 2/3 weekly individual sessions lasting 45 to 60 minutes, a weekly session for parents was also planned	Intervention group shows significantly higher decrease in scales related to problem behavior and emotional problems (withdrawn, somatic complaints, attention problems, social problems, internalizing problems, total problems) and significantly higher increase in a social skills scale than control group
Cognitive, Learning, and Problem-solving Skills Training	1. Patel et al., 2009 [64]; 2. USA	1. Uncontrolled intervention study (intervention group; pre, post); 2. n = 12 survivors	1. M = 5.96 years (SD = 4.86); 2. M = 7.23 years (SD = 2.75); 3. N/A	Children, central nervous system-involved cancer diagnosis, a minimum of 6 months of posttreatment completion with stable medical status, minimum age of 7 years	15 consecutive weekly 60 to 90-minute child-training sessions	Significant positive changes in social skills, significant positive changes in externalizing problems; No significant changes in the internalizing score and the attention subscale
Computer-Mediated Support Group Intervention (CMSG)	1. Bragadottir, 2008 [65]; 2. Iceland	1. Uncontrolled intervention study (intervention group; pre, 2 months after initiation, post); 2. n = 21 parents of 13 children	1. The majority of the children had been diagnosed at the age of 5 years or younger (n = 9); 2. 2 to 6 years; 3. N/A	Parents of children who were diagnosed with cancer at the age of 18 years or younger, and had completed their treatment within the past 5 years before the study	Computer-mediated support group, list-serve or mailing list, where participants used their own e-mail addresses for sending and receiving messages; Group discussion was unstructured, 4 consecutive months	Mothers’ depression decreased significantly from the second measurement time point to post; Fathers’ anxiety decreased significantly from pre to post; Fathers’ stress decreased significantly from the second measurement time point to post; Mothers and fathers perceived mutual support to some extent from participating in the CMSG
Cope, Adapt, Survive: Life after CAncEr (Cascade)	1. Wakefield et al., 2016 [66]; 2. Australia	1. Randomized controlled trial (intervention group, 6-month waitlist control group; baseline, 2 weeks post intervention, 6 months post intervention); 2. n = 56 parents from n = 54 families randomized (efficacy analysis 6 months post intervention: n = 19 intervention group, n = 16 waitlist)	1. Intervention group M = 5.22 years (SD = 4.24), waitlist group M = 5.72 years (SD = 4.20); 2. Intervention group: M = 2.93 years (SD = 2.14), waitlist group: M = 2.25 years (SD = 1.06); 3. N/A	Parents with a child aged 15 years or younger who had completed cancer treatment with curative intent in the past 5 years	Manualized program with 3 weekly 2-hour online sessions, synchronous e-mental health intervention delivered "live" by a psychologist in real time	No significant main effect of group or time on quality of life, psychological functioning, and family functioning; Significant main effect of time on the fear of cancer recurrence for both groups (significantly lower 2 weeks and 6 months post intervention)
Education-based and Peer discussion-based Group Interventions	1. Zhu et al., 2015 [67]; 2. China	1. Randomized controlled trial (education-based group (EG), peer discussion-based group (PDG); 4 time points: baseline, posttest, 2-week follow up, 6-month follow up); 2. n = 45 survivors (n = 22 EG, n = 23 PDG)	1. EG: M = 7.0 years; PDG: M = 6.4 years; 2. N/A; 3. N/A	Survivors, medulloblastoma diagnosis, 9 to 18 years old	Weekly group sessions for 8 consecutive weeks; EG and PDG lasted for 45 and 60 minutes respectively	Survivors in both groups improved over time; At posttest and 2-week follow-up, children in PDG scored lower on the perceived competence total score as compared to subjects in EG; EG scored significantly lower on physical appearance and considerably higher on global self-worth and social acceptance; Controlling for a time effect, the substantial group-by-time interaction suggested that the EG intervention had a positive effect on social acceptance, global self-esteem and behavioral conduct
Family Oriented Rehabilita-tion	1. Däggelmann et al., 2017 [58]; 2. Germany	1. Non-randomized controlled intervention study (survivors, siblings; pre, post, 6-month follow-up); 2. n = 22 patients, n = 20 siblings	1. N/A; 2. N/A; 3. N/A	Families of childhood cancer patients after cancer treatment; Focus on patients and siblings	Inpatient rehabilitation program for the whole family; Several sports activities	Significant improvement of the global quality of life score of patients from pre to follow-up; Significant improvement in the subscale mental well-being in patients from pre to post; Significant improvements in fatigue in patients and siblings
	1. Häberle et al., 1991 [55]; 2. Germany	1. Uncontrolled intervention study (intervention group; pre, post); 2. n = 44 families	1. N/A; 2. N/A; 3. N/A	Families of childhood cancer patients after cancer treatment	Inpatient rehabilitation program for the whole family; Interdisciplinary, family-oriented, psychosocial program	Child report and parent proxy: Significant improvement of the physical and psychological situation; Parent report: Significant positive changes in psychosomatic problems and in depressive symptoms, significant improvement in partner relationship
	1. Häberle et al., 1997 [56]; 2. Germany	1. Non-randomized controlled intervention study (intervention group, waitlist group; pre, post); 2. n = 104 families	1. N/A; 2. M = 18.2 months (SD = 13.91); 3. N/A	Families of childhood cancer patients after cancer treatment	4-week inpatient rehabilitation program for the whole family; Somatic, psychological and social rehabilitation	Behavioral problems in patients and siblings decreased significantly from pre to post; Significant reduction in the severity of physical and psychological symptoms in parents; Parents in the intervention group post significantly better than waitlist group pre
	1. Inhestern et al., 2017 [59]; 2. Germany	1. Uncontrolled intervention study (intervention group; pre, post); 2. n = 69 parents from n = 49 families	1. N/A; 2. M = 24.7 months (SD = 30.2); 3. M = 9.7 months (SD = 19.2)	Families of childhood cancer patients after cancer treatment; Focus on parents	Inpatient rehabilitation program for the whole family, group/couple/individual therapy, nutrition counselling, sports activities	Significant improvement in the anxiety and depression scores in parents from pre to post, no significant effect for gender and no significant interaction effect for time and gender
	1. van Buiren et al., 1998 [57]; 2. Germany	1. Uncontrolled intervention study (intervention group; pre, post, follow up 6.5 years after rehabilitation; in this paper focus on follow up); 2. n = 49 families pre, post, n = 24 families follow up	1. N/A; 2. N/A; 3. N/A	Families of childhood cancer patients	4-week inpatient rehabilitation program	At follow-up no significant differences to the general public; Psychological burden in parents and children decreased from pre to follow-up in ‘normal’ families and increased from pre to follow-up in highly burdened families
FAMily-Oriented Support (FAMOS)	1. Salem et al., 2017 [68]; 2. Denmark	1. Feasibility of a randomized controlled trial (intervention group, control group; post); 2. n = 57 families (n = 30 intervention group, n = 27 control group)	1. N/A; 2. 60% of the survivors between 1 and 2 years after diagnosis; 3. N/A	Survivors, cancer diagnosis, 0 to 6 years old (children who did not receive chemotherapy or radiotherapy were eligible up to the age of 18 years), the required time since the end of intensive cancer treatment depends on the cancer type; Parents and siblings	Home-based; Up to 6 sessions over a 6-month period, 3 sessions for parents, 2 for children (7 years or older) and one booster session for the whole family; 2 additional sessions for parents of families with children under the age of 7 on teaching the techniques to their children	Only assessment of satisfaction with the intervention; No psychosocial outcome parameters assessed
Group Social Skills Intervention Program (SSKIP)	1. Barrera & Schulte, 2009 [47]; 2. Canada	1. Uncontrolled intervention study (intervention group; baseline, pre, post); 2. n = 32 children and adolescents	1. M = 7.31 years; 2. M = 6.28 years (SD = 3.94); 3. M = 5.30 years (SD = 4.12);	Children and adolescents, brain tumor diagnosis, on follow-up care after the end of treatment, 8 to 18 years old	2-hour weekly group sessions for 8 weeks	Child report: No significant pre-post changes over time in any outcomes; No significant follow up changes for any of the outcomes; Parent report: Significant improvements in social skills, self-control, quality of life (in children); Significant time main effect for total behavioral problems but post-hoc analyses did not show any significant pre-post changes
	1. Barrera et al., 2017 [48]; 2. Canada	1. Randomized controlled trial (intervention group, attention placebo group; baseline, end of intervention, 6-month follow up); 2. n = 91 patients (n = 43 intervention group, n = 48 placebo group)	1. N/A; 2. Intervention group: M = 5.70 years (SD = 3.17), control group: M = 4.35 years (SD = 2.80); 3. Intervention group: M = 4.76 years (SD = 3.12), control group: M = 3.52 years (SD = 2.66)	Children and adolescents, brain/spinal tumor diagnosis, off treatment for at least 3 months, 8 to 16 years old	2-hour weekly group sessions for 8 weeks; Games and crafts, snack time, homework, and a graduation ceremony in both groups; Manualized SSKIP Program in the intervention group	Child report: Significantly higher social skills scores in the intervention group than in the control group; Children in the intervention group with a low social skills score at baseline showed the greatest improvements that persisted at follow up; Significantly higher empathy subscale scores in the intervention group compared to controls; No significant differences regarding quality of life; Caregiver and teacher report: No intervention effect
	1. Schulte, Bartels, & Barrera, 2014 [49]; 2. Canada	1. Non-randomized controlled intervention study (intervention group, control group; pre, post); 2. n = 27 survivors (n = 15 intervention group, n = 12 control group)	1. M = 6.81 years (SD = 3.18); 2. M = 5.51 years (SD = 2.35); 3. M = 4.20 years (SD = 2.27)	Survivors, central nervous system tumor diagnosis, off treatment and medically stable, 7 to 18 years old	8 group sessions	Child report: Significant reduction of social problems for the intervention group; Parent report: Significant improvements in social skills in the intervention group but not in the control group (in children), significant increase of social problems in the control group but not in the intervention group; Teacher report: Significant improvement in social skills for the intervention group (in children)
	1. Schulte, Vannatta, & Barrera, 2014 [50]; 2. Canada	1. Uncontrolled intervention study (intervention group; pre, post); 2. n = 15 survivors	1. M = 6.62 years (SD = 2.95); 2. M = 5.15 years (SD = 2.12); 3. M = 3.94 years (SD = 1.96)	Survivors, brain tumor diagnosis, completed treatment and medically stable, 7 to 18 years old	2-hour weekly group sessions for 8 weeks	No significant changes in social problem solving; Significant increases were found in social performance (Frequency of maintaining eye contact, social conversations with peers and off-task behavior (solitary activity)), listening to other speak decreased
Hope Therapy	1. Shekarabi-Ahari et al., 2012 [69]; 2. Iran	1. Randomized controlled trial (experimental group, control group; pre, post, follow up; follow up only in experimental group); 2. n = 20 mothers (n = 10 experimental group, n = 10 control group)	1. N/A; 2. N/A; 3. N/A	Mothers whose children suffered from cancer, depression score higher than 19 in Beck’s Depression Inventory and hope score less than 20 in Snyder Hope Scale	8 weekly 2-hour group sessions	Hope score increased significantly and depression score decreased significantly, Follow up results showed no significant changes in hope but depression decreased significantly in the experimental group
Integrated Adventure-Based Training and Health Education Program	1. Li et al., 2013 [70]; 2. China	1. Randomized controlled trial (experimental group, placebo control group; time of recruitment, 3 months after starting the intervention, 6 months after start, 9 months after start); 2. n = 71 children	1. N/A; 2. N/A; 3. between 6 and > 60 months	Survivors, cancer diagnosis, completed treatment at least 6 months previously, 9 to 16 years old	4-day integrated adventure-based training and health education program with activities such as educational talks, a workshop to develop a feasible individual action plan for regular physical activity, and adventure-based training activities	Quality of life increased significantly from the time of recruitment to nine months after the start in the experimental group but not in the control group; Physical activity and self-efficacy increased in both groups significantly
Oncology Camp/ Summer Camp	1. Conrad & Altmaier, 2009 [71]; 2. USA	1. Uncontrolled cross-sectional intervention study (intervention group; post intervention); 2. n = 26 families responded, final sample n = 25	1. M = 6.67 years (SD = 5.27); 2. N/A; 3. n = 17 in remission, n = 3 active treatment	Children, cancer diagnosis, currently on treatment or in remission, 5 to 18 years old	Week-long camp, common camp activities	Campers reported receiving more support at camp than children in the general population; No significant differences between means for internalizing, externalizing, and total standard scores from a behavioral problem scale in the camp sample in comparison to the general population or the clinical sample
Oncology Summer Camp	1. Meltzer & Rourke, 2005 [72]; 2. USA	1. Uncontrolled intervention study (intervention group; pre, post); 2. n = 34 adolescents	1. N/A; 2. M = 8.7 years; 3. M = 5.6 years	Adolescents, cancer diagnosis	Week-long summer camp	From pre to post no significant changes in the loneliness scores; No pre-post comparisons reported for the other outcomes
Op Koers Oncologie (OK Onco)	1. Maurice-Stam et al., 2009 [73]; 2. Netherlands	1. Uncontrolled intervention study (intervention group; pre, post); 2. n = 11 children	1. N/A; 2. N/A; 3. 1 to 6 years	Children, cancer diagnosis, completed treatment successfully, 8 to 12 years old	Six psycho-educational group sessions	Several children showed improvements on some items; No p-values reported, only score differences pre to post
Op Koers Oncologie Online (OK Onco Online)	1. Maurice-Stam et al., 2014 [74]; 2. Netherlands	1. Uncontrolled cross-sectional intervention study (intervention group; post intervention); 2. n = 12 children, n = 11 completed the questionnaire	1. N/A; 2. 2 to 6 years (1 participant 14 years); 3. N/A	Adolescents, completed treatment successfully, 12 to 18 years old	Online cognitive behavioral based group intervention, 6 structured weekly chat sessions of 90 minutes each with home exercises, after 6 months a booster session of 90 minutes; The website also serves as a source of information for siblings and parents	Only assessment of satisfaction and feasibility of OK Onco Online; No psychosocial outcome parameters assessed
Peer-Mediated Intervention	1. Devine et al., 2016 [75]; 2. USA	1. Non-randomized controlled intervention study (4 groups: intervention/control x brain tumor survivors/classroom participants; entry into the study, end of the school year); 2. n = 12 patients (8 intervention, 4 control); classroom final sample n = 269 (136 intervention, 81 control)	1. N/A; 2. Intervention group: M = 4.5 years (SD = 2.7), control group: M = 5.2 years (SD = 1.9); 3. Intervention group: M = 2.8 years (SD = 2.2), control group: M = 4.1 years (SD = 1.4)	Survivors, brain tumor diagnosis, no evidence of active disease, at least 3 months beyond completion of therapy, in a regular classroom at least part of the day, 6 to 14 years old; Healthy peers	Small group sessions for survivors and healthy peers: Between 5 and 8 sessions, 30 to 40 minutes each, occurring twice weekly within one school year; The brain tumor survivor was never identified	Survivors in intervention classrooms had on average about 2 more friendship nominations at the end of the school year than those in control classrooms; No differences in social acceptance, social rejection or victimization between the groups; Intervention classrooms demonstrated significantly lower levels of social rejection and victimization than comparison classrooms at the end of the school year
Quality of Life in Motion (QLIM)	1. van Dijk-Lokkart et al., 2015 [53]; 2. Netherlands	1. Part of a randomized controlled trial; in this study only results of the intervention group (intervention group; pre, post); 2. n = 30 patients	1. N/A; 2. M = 1.07 years (SD = 0.11); 3. N/A	Patients, cancer diagnosis, treated with chemotherapy and/or radiotherapy, were no longer than 12 months off treatment, 8 to 18 years old; Their parents	12 weeks, 6 child sessions of 60 minutes each scheduled once every 2 weeks, at the start and the end of the program a parent session is included, children are not present during these 2 parent sessions	Only assessment of feasibility and satisfaction with the psychosocial training; No psychosocial outcome parameters assessed
	1. van Dijk-Lokkart et al., 2016 [54]; 2. Netherlands	1. Randomized controlled trial (intervention group, control group; baseline, 4 months after baseline, 12 months after baseline); 2. n = 68 patients (n = 30 intervention group, n = 38 control group)	1. N/A; 2. N/A; 3. N/A	Patient, cancer diagnosis, treated with chemotherapy and/or radiotherapy, and were no longer than 12 months off treatment, 8 to 18 years old; Their parents	Combined physical exercise and psychosocial training, 12 weeks; Physical exercise: 2 sessions per week of 45 minutes each in a local physiotherapy practice; Psychosocial training: 6 child sessions of 60 minutes each, scheduled once every 2 weeks, at the start and the end of the program a parent session is included	Child report: No significant differences between groups; Parent report: Greater improvement on pain-related health-related quality of life on short-term and long-term, greater improvement on procedural anxiety on the short-term and on nausea long-term in the intervention group (in children)
Siblings Coping Together (SibCT)	1. Salavati et al., 2014 [76]; 2. Canada	1. Uncontrolled intervention study (intervention group; pre, post); 2. n = 151 siblings, database n = 111	1. N/A; 2. N/A; 3. N/A	Children who had/have a brother/sister who was currently treated for cancer and at least 3 months post diagnosis or had finished treatment within the last year, 8 to 17 years old	8 weekly 2-hour manualized group therapy sessions with 4 to 7 siblings	Greater improvement of anxiety and depression symptoms post-intervention in the less resilient group than in the more-resilient group; Within the less resilient group, siblings of brain tumor patients improved less in their depression scores than siblings of children with other cancer diagnoses
Social-Skills Training Group Intervention	1. Barakat et al., 2003 [77]; 2. USA	1. Uncontrolled intervention study (intervention group; baseline, follow up); 2. n = 18 families	1. M = 4.82 years (SD = 3.24); 2. N/A; 3. M = 4.22 years (SD = 2.72)	Children, received brain tumor treatment, off treatment for at least 6 months, 8 to 14 years old; Parents of these children	Children’s group and parent’s group, 6 weekly sessions, weekly homework, final session together	Child report: Significant positive changes in internalizing, externalizing and social competence; Parent report: Significant positive changes in total competence (in children); Teacher report: Significant positive changes in externalizing and problem behaviors (in children)
Surviving Cancer Competently Intervention Program (SCCIP)	1. Kazak et al., 1999 [51]; 2. USA	1. Uncontrolled intervention study (intervention group; pre, 6 months post); 2. n = 19 families	1. N/A; 2. N/A; 3. N/A	Families of adolescent patients, had completed their cancer treatment at least one year previously	1-day group workshop with 4 sessions	Symptoms of posttraumatic stress and anxiety decreased, changes in family functioning could be found only in some areas of family functioning (no p-values reported)
	1. Kazak et al., 2004 [52]; 2. USA	1. Randomized controlled trial (intervention group, waitlist control group; baseline, approximately 3 to 5 months post intervention); 2. n = 150 families	1. M = 7.80 years; 2. N/A; 3. M = 5.30 years (SD = 2.92)	Survivors, cancer diagnosis, completed cancer treatment 1 to 10 years previously, 11 to 19 years old; Their families	4-session, 1-day manualized group intervention that integrates cognitive behavioral treatment with family therapy	In comparison to the control group, significant reductions in arousal among survivors and in intrusive thoughts among fathers in the intervention group
The Home-Based Aerobic Exercise Intervention	1. Yeh et al., 2011 [78]; 2. Taiwan	1. Non-randomized controlled intervention study (intervention group, control group; 8 time points: pretest, once weekly during the intervention (5 weeks), posttest, 1-month follow up); 2. n = 24 patients participated, n = 22 final sample (n = 12 intervention group, n = 10 control group)	1. N/A; 2. N/A; 3. N/A	Children and adolescents diagnosed with acute lymphoblastic leukemia, treated with chemotherapy	6-week home-based aerobic exercise intervention, 3 days a week, for 30 minutes each session, video guide	Intent to treat analyses: No intervention and time effects for any of the 3 fatigue subscales at any points of time; Per protocol analyses: Children in the intervention group reported significantly lower general fatigue than those in the control group at the 1-month follow-up measurement
Yoga Intervention	1. Hooke et al., 2016 [79]; 2. USA	1. Uncontrolled intervention study (intervention group; 6 weeks pre intervention, before first session, after sixth yoga session); 2. n = 13 children and adolescents	1. N/A; 2. N/A; 3. M = 10.5 months (SD = 5.73)	Children and adolescents, completed treatment in the past 2 to 24 months for a pediatric cancer (leukemia, lymphoma, solid tumor, and/or central nervous system (CNS) tumor), received chemotherapy, radiation, or (for CNS tumor patients only) surgery, 10 to 18 years old	6 weekly yoga classes in one of the study institutions, DVD to practice yoga in the home setting twice a week	Only children (n = 7) and not adolescents had a significant decrease in their anxiety score

#### Study characteristics

The included studies were published between 1991 [55] and 2017 [48, 58, 59, 68]. The majority of the studies was conducted in North America or Europe (85%, n = 28) [47–65, 68, 71–77, 79]. Most of the studies were intervention studies without a control group and with at least two measurement time points (n = 15) [47, 50, 51, 53, 55, 57, 59, 61, 64, 65, 72, 73, 76, 77, 79]. Eight papers reported randomized controlled trials [48, 52, 54, 66–70], six papers described non-randomized controlled intervention studies [49, 56, 58, 63, 75, 78], and four papers reported uncontrolled cross-sectional intervention studies [60, 62, 71, 74].

In most studies (n = 15), the childhood cancer patient was the target person of the intervention [47–50, 63, 64, 67, 70–75, 78, 79]. The remaining studies describe interventions for the family (n = 9) [51, 52, 55–59, 62, 68], for the patient and the parents (n = 4) [53, 54, 60, 77], for the parents only (n = 3) [65, 66, 69], and for the siblings only (n = 1) [76] or the siblings and the patient (n = 1) [61]. More than half of the studies describe interventions for patients or family members of patients with any type of childhood cancer (67%, n = 22) [51–59, 61, 62, 65, 66, 68–74, 76, 79]. Thirty percent of the papers (n = 10) evaluate studies for CNS tumor patients or their family members [47–50, 60, 63, 64, 67, 75, 77] and one study focusses on leukemia [78]. Most of the studies focus on interventions for children from approximately 8 to 18 years (76%, n = 25). Four studies focus primarily on younger children [66, 68, 69, 73], and four studies on adolescents [51, 52, 72, 74].

The studies cover a wide range of different intervention settings. Most of the studies describe interventions conducted in an outpatient group setting (n = 15) [47–52, 60, 67, 69, 70, 73, 75–77, 79]. The other studies describe inpatient rehabilitation programs (n = 5) [55–59], interventions in an outpatient individual setting (n = 4) [53, 54, 63, 64], interventions in a camp setting (n = 4) [61, 62, 71, 72], computer-based interventions (n = 3) [65, 66, 74], and home-based interventions (n = 2) [68, 78].

The primary aims of the interventions vary across the studies. One study compared two interventions, one with a psychoeducational focus and one focusing on social support [67]. Therefore, we categorized 34 interventions from 33 studies regarding their primary aim: Reduction of psychological burden (n = 9) [51–53, 63, 66, 68, 69, 74, 76], reduction of physical and psychological burden (n = 9) [54–59, 70, 78, 79], improvement of social skills (n = 8) [47–50, 60, 64, 75, 77], increase of social support (n = 6) [61, 62, 65, 67, 71, 72], and psychoeducation (n = 2) [67, 73].

#### Synthesis of the psychosocial effects of the interventions

In the following, we summarize the study results of the 28 papers describing effects on psychosocial outcome parameters briefly and synthesize the findings according to the setting of the interventions. In five studies, the authors only assessed the participants’ satisfaction and the feasibility of the interventions and no psychosocial outcome parameters [53, 60, 62, 68, 74]. A comprehensive overview of the interventions and study characteristics is provided in Table 1.

15 studies describe interventions in an *outpatient group setting* [47–52, 60, 67, 69, 70, 73, 75–77, 79]. Most of these interventions target childhood cancer survivors and cover a wide range of different programs (e.g. yoga program [79], psycho-education [73]). The studies reported a positive impact of these interventions on social skills [75], anxiety scores [79], quality of life [70], and other psychosocial variables [67, 73], partly with mixed results [47–50]. We also identified outpatient group interventions for families, parents and siblings. Studies on interventions for survivors and parents or the family as a whole reported significant positive changes in various psychosocial outcome parameters (e.g. social skills, posttraumatic stress) in children and their family members [51, 52, 77]. We identified one outpatient group intervention for mothers only [69]. The maternal hope score increased and the depression score decreased significantly. Further, a paper evaluating an outpatient group intervention for siblings found a reduction of anxiety and depression symptoms post intervention, in particular in the less resilient group [76].

Four different cancer *camps* were evaluated in the included studies [61, 62, 71, 72]. Cancer camps had a positive impact on social support in childhood cancer patients and survivors [71] as well as on the independence, social skills, and self-esteem of survivors and siblings [61]. The evaluation of a camp for adolescents with cancer could not reveal any changes in loneliness scores from pre to post [72].

Five studies evaluated a *family oriented rehabilitation program* [55–59]. A positive impact of the rehabilitation program on the physical and psychological situation of patients, siblings and parents as well as improvements in partner relationship could be found from pre to post and in comparison to a comparison group [55, 56, 58, 59]. At 6.5 years follow up, the psychosocial situation of families participating in the rehabilitation program did not significantly differ from the situation of families from the general public [57].

Three studies evaluated *computer-based interventions* and generally found mixed evidence [65, 66, 74]. Whereas a computer-based support group intervention for parents led to a significant decrease of maternal depression and paternal anxiety and stress [65], an e-mental health intervention for parents did not improve parental quality of life, psychological functioning, and family functioning [66].

Four studies described *outpatient individual interventions* [53, 54, 63, 64]. A physical and psychosocial intervention for children and their parents did not lead to an improvement of psychosocial outcomes with some exceptions for parent-reported subscales [54]. In contrast, interventions for brain tumor survivors had a positive impact on psychological outcome parameters [63] as well as social skills [64].

Only one study assessed psychosocial outcomes in participants of a *home-based intervention* [78]. The authors evaluated an aerobic intervention for children and adolescents with acute lymphoblastic leukemia and could not reveal any significant intervention or time effects for any fatigue subscale and measurement time points in the intent-to-treat analysis [78]. However, the per-protocol analysis revealed significantly lower general fatigue in the intervention group than in the comparison group at 1-month follow up.

### Risk of bias

The quality of all studies was assessed with the EPHPP Quality Assessment Tool [43, 44]. Most of the studies received the final grade weak (n = 19). Another 13 studies were considered moderate and only one study received the final grade strong. The most frequent weak ratings were assigned in the categories selection bias (n = 19) and data collection methods (n = 22), whereas only five studies received the rating weak for their study design (Table 2). In the EPHPP Quality Assessment Tool, the selection bias is determined by the representativeness and the participation rate. Most of the studies reported a participation rate below 60% (n = 12 studies). In nine studies, participation rates were not reported at all. In six studies, 60% to 79% of the patients or family members participated and in six studies, the reported participation rates were between 80% and 100%. In eight studies, the authors compared nonparticipants with participants. They found no significant differences regarding demographic characteristics (e.g. age, ethnicity, gender). However, there were significant differences in the diagnoses of the children [48]. One study additionally found differences in the fitness level and internalizing behavioral problems [54]. Participants reported a lower fitness level and more internalizing behavioral problems than nonparticipants did.

**Table 2:** Methodological quality assessment of the included studies (N = 33) with the Effective Public Health Practice Project Quality Assessment Tool.

Study	Selection bias	Design	Confounders ^a^	Blinding ^a^	Data collection methods	Withdrawals and drop-outs ^b^	Global rating
Barakat et al., 2003 [77]	Weak	Moderate	N/A	N/A	Strong	Moderate	Moderate
Barrera & Schulte, 2009 [47]	Moderate	Moderate	N/A	N/A	Weak	Strong	Moderate
Barrera et al., 2017 [48]	Weak	Strong	Moderate	Moderate	Strong	Strong	Moderate
Bragadottir, 2008 [65]	Weak	Moderate	N/A	N/A	Weak	Moderate	Weak
Conrad & Altmaier, 2009 [71]	Weak	Weak	N/A	N/A	Weak	N/A	Weak
Däggelmann et al., 2017 [58]	Weak	Moderate	Moderate	Moderate	Strong	Moderate	Moderate
Dawson et al., 2012 [61]	Moderate	Moderate	N/A	N/A	Weak	Weak	Weak
Devine et al., 2016 [75]	Moderate	Moderate	Weak	Moderate	Weak	Strong	Weak
Die-Trill et al., 1996 [60]	Moderate	Weak	N/A	N/A	Weak	N/A	Weak
Häberle et al., 1991 [55]	Moderate	Moderate	N/A	N/A	Weak	Weak	Weak
Häberle et al., 1997 [56]	Moderate	Moderate	Weak	Moderate	Weak	Weak	Weak
Inhestern et al., 2017 [59]	Moderate	Moderate	N/A	N/A	Weak	Strong	Moderate
Hooke et al., 2016 [79]	Weak	Moderate	N/A	N/A	Weak	Moderate	Weak
Kazak et al., 1999 [51]	Weak	Moderate	N/A	N/A	Strong	Strong	Moderate
Kazak et al., 2004 [52]	Weak	Strong	Strong	Moderate	Strong	Moderate	Moderate
Li et al., 2013 [70]	Moderate	Strong	Strong	Moderate	Strong	Strong	Strong
Maurice-Stam et al., 2009 [73]	Weak	Moderate	N/A	N/A	Weak	Weak	Weak
Maurice-Stam et al., 2014 [74]	Weak	Weak	N/A	N/A	Weak	N/A	Weak
Meltzer & Rourke, 2005 [72]	Moderate	Moderate	N/A	N/A	Weak	Strong	Moderate
Patel et al., 2009 [64]	Weak	Moderate	N/A	N/A	Weak	Strong	Weak
Poggi et al., 2009 [63]	Moderate	Moderate	Strong	Weak	Strong	Weak	Weak
Salavati et al., 2014 [76]	Weak	Moderate	N/A	N/A	Weak	Weak	Weak
Salem et al., 2017 [68]	Moderate	Strong	Weak	Moderate	Weak	Strong	Weak
Schulte, Bartels, & Barrera, 2014 [49]	Weak	Strong	Strong	Moderate	Strong	Weak	Moderate
Schulte, Vannatta, & Barrera, 2014 [50]	Weak	Moderate	N/A	N/A	Weak	Strong	Weak
Shekarabi-Ahari et al., 2012 [69]	Weak	Strong	Strong	Moderate	Moderate	Weak	Weak
van Buiren et al., 1998 [57]	Weak	Moderate	N/A	N/A	Weak	Weak	Weak
van Dijk-Lokkart et al., 2015 [53]	Weak	Weak	N/A	N/A	Weak	N/A	Weak
van Dijk-Lokkart et al., 2016 [54]	Weak	Strong	Strong	Moderate	Strong	Moderate	Moderate
Wakefield et al., 2016 [66]	Moderate	Strong	Strong	Moderate	Weak	Moderate	Moderate
Wu et al., 2011 [62]	Weak	Weak	N/A	N/A	Weak	N/A	Weak
Yeh et al., 2011 [78]	Moderate	Moderate	Strong	Moderate	Strong	Strong	Moderate
Zhu et al., 2015 [67]	Moderate	Strong	Strong	Moderate	Weak	Strong	Moderate

^a^ For studies with only one group confounders and blinding was set N/A;

^b^ For studies with only one measurement time point withdrawals and drop-outs was set N/A

## Discussion

We identified 33 studies evaluating psychosocial interventions for childhood cancer survivors and/or their family members during the first five years after the end of cancer treatment. The investigated interventions show a heterogeneity of aims, target persons and settings.

Most of the investigated interventions aim to improve psychological burden. Others aim to improve social skills or provide social support and psychoeducation. Besides, nine interventions aim to improve both the physical and psychological situation [54–59, 70, 78, 79]. These results are consistent with a review analyzing the association between physical exercise and well-being in several subgroups (e.g. adult cancer patients), which found a positive impact of exercise and physical interventions on mental well-being in adult cancer patients [80]. Another review investigated physical exercise training interventions for children and young adults during and after cancer treatment [81]. The authors could not reveal any positive impact of exercise on well-being [81]. In this review, we found mixed results on the impact of physical activity interventions or combined physical and psychosocial interventions on mental health [54–59, 70, 78, 79]. However, we only included studies with a psychosocial focus and other studies on physical interventions for childhood cancer may have been omitted.

In our systematic review, we analyzed studies evaluating interventions for survivors, siblings, parents, and for the whole family. Numerous studies have shown that some siblings experience high levels of psychosocial burden [17]. Even though siblings of long-term survivors do not report elevated distress levels per se, a childhood cancer disease can be a risk factor for long-term distress in some siblings [82]. Social support seems to be a protective factor in siblings of childhood cancer patients and thus psychosocial interventions focusing on social support could prevent long-term negative psychosocial consequences. However, only two of the included studies investigated interventions addressing siblings [61, 76]. Furthermore, the family as a whole has to adapt to the illness related changes. A recent systematic review revealed a significant association between family functioning and child adjustment [83]. Therefore, psychosocial support for the family as a whole is highly recommended. Only 9 of the 33 included studies investigated interventions for the family as a whole [51, 52, 55–59, 62, 68]. Most of the studies focused on children from approximately 8 to 18 years, while younger children and their family members received less attention. After treatment, also preschool children have a lower health-related quality of life than their healthy peers [84]. Their scores seem to normalize after two to three years after treatment. However, the time from the end of cancer treatment to normalcy can be very challenging and thus psychosocial support is nevertheless recommended [84].

The included studies investigate interventions in various unconventional child- and family-oriented settings. Among others, we identified studies investigating camps, computer-based interventions and half of the included studies evaluated group interventions. This indicates that different attempts have been made to improve the psychosocial situation of affected families. Even though clinical efficacy could not be confirmed in all of the included studies, for all settings at least some studies revealed a statistically significant benefit and therefore offer starting points for further research. It is important to mention, that the high heterogeneity of different interventions and settings might also be a result of differences in health care systems in the different countries. For instance, the family oriented rehabilitation program is a special feature of the German health care system.

Overall, the methodological quality of the included studies assessed with the EPHPP Quality Assessment Tool was rather poor. Only one study showed a strong quality and the majority of the studies received the final grade weak. Especially high selection bias and poorly described data collection methods caused unsatisfactory final grades. Only five studies received the grade weak for their study design and thus many of the included studies applied appropriate designs and methods but lacked adequate reporting. Some studies used validated and reliable measurement scales but did not describe their psychometric properties. Since the EPHPP Quality Assessment Tool Dictionary does not provide unambiguous recommendations on this topic [85], we decided to rate studies as strong in this component if they described the reliability and validity of the instruments or referred to their study protocol in which they had described the psychometric properties. Other studies did not describe the blinding nor did they report drop-out rates. However, it is very likely that small sample sizes and a high selection bias are consequences of this particular population. The recruitment of childhood cancer survivors and their families can be difficult. First, childhood cancer is a rare disease [1]. Second, even after successful treatment of the cancer disease, survivors as well as parents and siblings can suffer from high levels of distress, which can reduce the willingness to participate [3, 11, 12, 17]. Only few studies provided information on nonparticipants. Participants and nonparticipants were compared with respect to demographic and medical characteristics (e.g. age, ethnicity, gender, diagnosis). Only one study compared the groups regarding their physical and psychological situation [54]. Due to the lack of information on nonparticipants, a self-selection bias cannot be ruled out and therefore the generalizability of the study results is limited [86]. Furthermore, many studies assessed multiple outcome parameters and did not adjust for multiple testing. Multiple testing leads to an type 1 error inflation [87]. Thus, the number of falsely rejected null hypotheses increases with the number of test hypotheses. With regard to the included evaluation studies, this means that the probability of finding false-positive effects of the investigated interventions increases with the inclusion of multiple outcome parameters and the omission of adjustment. Therefore, it is difficult to draw a conclusion on the efficacy of the investigated interventions based on the included studies. Additionally, the assessment of multiple outcomes within and across the studies contributes to difficulties in the aggregation of study results. Four studies had a cross-sectional design [60, 62, 71, 74]. More than half of the included studies did not have a control or comparison group. Only 8 of the 33 included studies are randomized controlled trials [48, 52, 54, 66–70]. Taking together these methodological limitations, the results of the studies can only be interpreted with caution. The effects of the interventions might have been overestimated in the included low quality studies. More high quality studies are necessary to validate prior findings.

There are several implications for research and clinical practice. From a health care research perspective, more high quality studies investigating the efficacy of psychosocial interventions for childhood cancer survivors and their family members are necessary. Only reliable studies can affect the psychosocial aftercare of survivors and their families substantially. Therefore, more high quality randomized controlled trials should be conducted. Additionally, future studies should counteract the low reporting quality by following reporting guidelines. Furthermore, siblings and the family as a whole should also be addressed in psychosocial interventions after the successful treatment of the patient. Overall, the investigated interventions helped families to improve their mental well-being and enhance social skills. These results can be used to optimize health care services that help families with the re-entry into daily life.

### Strengths and limitations

This systematic review has some limitations on study level and at review level. Firstly, most of the studies were conducted in North America and Europe. Therefore, the results cannot be generalized to other parts and cultures of the world. Moreover, we included only studies in English or German. Consequently, we may have overlooked studies in other languages. Due to the methodological heterogeneity of the included studies, we could not conduct a quantitative synthesis of the study results. Lastly, we conducted the systematic database search in four databases that are relevant for this field of research and conducted additional hand searches. We had to add only few studies to the records identified through our database search. Nevertheless, relevant studies published in peer-reviewed journals that are not covered by these databases, might have been missed.

This systematic review has also several strengths. First of all, we published a review protocol in the International prospective register of systematic reviews (PROSPERO) to conduce to transparency. Further, this review was conducted and reported according to the PRISMA checklist [35]. Two independent raters assessed the inclusion and exclusion criteria to increase objectivity. Finally, we conducted a quality assessment of the included studies to ensure the reliability of the results.

## Conclusions

In conclusion, this review provides a comprehensive overview of existing interventions for childhood cancer patients and their family members. However, the high heterogeneity of addressees, methods and outcomes calls for further systematic research. To our knowledge, this is the first systematic review focusing on the vulnerable time after cancer treatment to five years later. Our results suggest that various interventions can help to improve the well-being of affected families. Still, more high quality studies are necessary to validate previous findings and to develop future comprehensive interventions. Finally, this systematic review gives implications for future research and may help to optimize health care services for childhood cancer survivors and their families.

## Supporting information

S1 Table PRISMA checklist. (DOC)

S1 File PROSPERO protocol. (PDF)
