# Supplementary material for: Psychosocial interventions for rehabilitation and reintegration into daily life of pediatric cancer survivors and their families: A systematic review
Source: PLoS One. 2018 Apr 19;13(4):e0196151. doi: 10.1371/journal.pone.0196151 (PMC5908186; doi:10.1371/journal.pone.0196151)
Supplement: S1 File — (PDF) [file pone.0196151.s002.pdf]

Psychosocial interventions for rehabilitation and reintegration into daily life of childhood cancer patients and their family members after the end of acute treatment: a systematic review

Mona Leandra Peikert, Laura Inhestern, Corinna Bergelt

Citation

Mona Leandra Peikert, Laura Inhestern, Corinna Bergelt. Psychosocial interventions for rehabilitation and reintegration into daily life of childhood cancer patients and their family members after the end of acute treatment: a systematic review. PROSPERO 2017 CRD42017059782 Available from: http://www.crd.york.ac.uk/PROSPERO/display_record.php?ID=CRD42017059782

Review question

1. Which psychosocial interventions for rehabilitation and reintegration into daily life of childhood cancer patients and their families do exist?
2. What are the effects of these interventions on psychosocial outcomes in the family members?

Searches

The following databases will be searched: CINAHL, MEDLINE, PSYNDEX, Web of Science.
Primary research articles in English and German will be included. There will be no restriction regarding the publication period. Additionally, hand searches of reference lists of relevant papers will be conducted.

Types of study to be included

Inclusion criteria:- quantitative, primary research- full text is accessible- article published in a peer-reviewed journal;Exclusion criteria:- qualitative research- conference proceedings.

Condition or domain being studied

This systematic review will focus on psychosocial interventions for childhood cancer patients and their family members after the end of acute treatment and their impact on various psychosocial outcome parameters.

Participants/population

Inclusion criteria: - Cancer patients and/or their family members
- Patients diagnosed with cancer before the age of 21;
Exclusion criteria:
- Primary focus on palliative treatment of the cancer patient.

Intervention(s), exposure(s)

Inclusion criteria:
- psychosocial intervention
- cancer specific intervention
- child-, family- or parent-focused intervention
- intervention takes place during the period from the end of acute treatment to five years later;
Exclusion criteria:
- intervention for children or family members of children with different chronic medical conditions
- intervention takes place during the acute treatment
- pharmacological, neurocognitive or educational interventions.

Comparator(s)/control

Comparators or control groups will not be required.

Primary outcome(s)

The primary outcomes are psychosocial interventions and psychosocial outcome parameters (e.g. depression, anxiety, quality of life, family functioning).

Secondary outcome(s)

None.

Data extraction (selection and coding)

Study selection:
1. Title and (in uncertainty) abstract screening by first author
2. Full text screening by two independent members of the research team;
Data extraction by two independent members of the research team:
Data to be extracted
- Authors, year, title, language, country
- Study design, sample size, patients age at diagnosis, time since diagnosis, time since end of acute treatment
- Name of intervention, target group, setting, psychosocial outcomes, effects of the intervention;
Disagreements between the two members of the research team will be resolved by consent after discussion.

Risk of bias (quality) assessment

The quality of the included studies will be assessed with the Effective Public Health Practice Project Quality Assessment Tool (EPHPP; Armijo-Olivo, Stiles, Hagen, Biondo, & Cummings, 2012; Thomas, Ciliska, Dobbins, & Micucci, 2004). Studies assessed with the EPHPP receive a final grade: strong, moderate or weak. The quality will be assessed independently by two raters. Disagreements between the raters will be resolved by consent after discussion.

Strategy for data synthesis

Due to the expected clinical, methodological and statistical heterogeneity of the studies, there will be no quantitative data synthesis. A narrative synthesis is planned (Dixon-Woods, Agarwal, Jones, Young, & Sutton , 2005).

Analysis of subgroups or subsets

None planned.

Contact details for further information

Mona Leandra Peikert
m.peikert@uke.de

Organisational affiliation of the review

University Medical Center Hamburg-Eppendorf, Department of Medical Psychology
https://www.uke.de/english/departments-institutes/institutes/medical-psychology/index.html

Review team members and their organisational affiliations

Ms Mona Leandra Peikert. University Medical Center Hamburg-Eppendorf, Department of Medical Psychology
Ms Laura Inhestern. University Medical Center Hamburg-Eppendorf, Department of Medical Psychology
Professor Corinna Bergelt. University Medical Center Hamburg-Eppendorf, Department of Medical Psychology

Anticipated or actual start date

01 November 2016

Anticipated completion date

31 October 2017

Funding sources/sponsors

None

Conflicts of interest
None known

Language
English

Country
Germany

Stage of review
Review_Completed_not_published

Subject index terms status
Subject indexing assigned by CRD

Subject index terms
Family; Humans; Life; Medicine; Neoplasms

Date of registration in PROSPERO
20 March 2017

Date of publication of this version
29 January 2018

Details of any existing review of the same topic by the same authors

Stage of review at time of this submission

Stage	Started	Completed
Preliminary searches	Yes	Yes
Piloting of the study selection process	Yes	Yes
Formal screening of search results against eligibility criteria	Yes	Yes
Data extraction	Yes	Yes
Risk of bias (quality) assessment	Yes	Yes
Data analysis	Yes	Yes

Versions
20 March 2017
29 January 2018

PROSPERO
This information has been provided by the named contact for this review. CRD has accepted this information in good faith and registered the review in PROSPERO. CRD bears no responsibility or liability for the content of this registration record, any associated files or external websites.
